# Recalibrating academic expertise in the age of generative AI

**DOI:** 10.1016/j.patter.2025.101473

**Published:** 2026-01-09

**Authors:** Zhicheng Lin, Aamir Sohail

**Affiliations:** 1 Department of Psychology, Yonsei University, Seoul 03722, Republic of Korea; 2 Department of Psychology, University of Science and Technology of China, Hefei 230026, China; 3 Centre for Human Brain Health, School of Psychology, University of Birmingham, Birmingham B15 2TT, UK; 4 Institute for Mental Health, School of Psychology, University of Birmingham, Birmingham B15 2TT, UK

## Abstract

The integration of generative AI (GenAI) into academic workflows represents a fundamental shift in scientific practice. While these tools can amplify productivity, they risk eroding the cognitive foundations of expertise by simulating the very tasks through which scientific competence is developed, from synthesis to experimental design to writing. Uncritical reliance can lead to skill atrophy and AI complacency. We propose a framework of essential AI meta-skills: strategic direction, critical discernment, and systematic calibration. These constitute a new form of scientific literacy that builds on traditional critical thinking. Through domain-specific examples and a pedagogical model based on situated learning, we show how these meta-skills can be cultivated to ensure that researchers, particularly trainees, maintain intellectual autonomy. Without deliberate cultivation of these meta-skills, we risk creating the first generation of researchers who serve their tools rather than direct them.

## The bigger picture

Throughout history, scientists have adopted new tools—from microscopes to statistical software—that transformed how research is conducted. Each tool required researchers to develop new competencies while retaining core scientific judgment. Generative AI (GenAI) represents a different kind of technological shift. Unlike previous tools that augmented specific capabilities, GenAI can perform the foundational intellectual activities through which scientific expertise itself develops: reading literature, designing studies, writing arguments, and generating code. This creates a paradox: the same technology that can accelerate research productivity also threatens to erode the cognitive skills that make researchers effective in the first place. The solution is not to reject AI tools but to cultivate a new form of scientific literacy. Researchers must learn how to strategically direct AI systems toward useful outputs, critically evaluate what these systems produce, and systematically verify results—all while maintaining intellectual ownership. This requires reconceptualizing scientific training to ensure that AI functions as cognitive scaffolding rather than a replacement for human reasoning. The decisions we make now about how researchers learn to work with AI will determine whether this technology amplifies scientific discovery or creates a generation dependent on systems they cannot meaningfully control.

## Teaser

GenAI tools promise accelerated discovery but risk eroding scientific expertise. When researchers delegate to AI, they bypass the effortful processes through which competence develops. This perspective proposes a framework of essential meta-skills—strategic direction, critical discernment, and systematic calibration—that constitutes a new form of scientific literacy. Through domain-specific examples and a situated learning model, we show how these capabilities can be cultivated to ensure AI amplifies human judgment and scientific autonomy.

## Introduction

For better or worse, generative AI (GenAI) has fundamentally altered scientific practice.^1^ Across disciplines, researchers increasingly deploy large language models (LLMs) for tasks once considered demonstrations of scientific skill: literature synthesis, experimental design, and manuscript writing.^2^^,^^3^ Analyses of the US labor market find that scientists and researchers face among the highest levels of task exposure to LLM-driven transformation.^4^

GenAI represents the latest step in AI’s emergence as a general method of invention^5^ in science. Over the past decade, AI has evolved from neural networks detecting patterns in complex datasets to machine learning models generating testable hypotheses across disciplines.^5^^,^^6^ GenAI crosses a critical threshold: autonomous hypothesis generation and discovery in constrained domains, or what some term the fifth era of science.^7^^,^^8^

Rather than augmenting specific analytical capabilities, GenAI simulates the broad cognitive activities through which academic expertise develops: synthesis, argumentation, and writing. This creates a paradox for scientific training. Tools that amplify productivity^9^ may simultaneously erode the cognitive foundation of competence.^10^ Moreover, when used for direct content generation, AI can diminish researchers’ sense of ownership, pride, and accountability for their work.^11^^,^^12^ Without specific training, researchers risk developing maladaptive practices, failing to engage with AI critically due to a lack of awareness, motivation, or ability.^13^

In this perspective, we argue that working effectively with GenAI while acquiring and maintaining scientific expertise requires developing essential AI meta-skills—a new form of scientific literacy comprising strategic direction, critical discernment, and systematic calibration. Through domain-specific examples and a pedagogical model based on situated learning, we propose how these meta-skills can be cultivated to ensure that researchers, particularly trainees, maintain intellectual autonomy. We demonstrate their necessity by highlighting concerning patterns of AI complacency: users progressively disengage from critical evaluation and develop ritualized acceptance of authoritative-seeming outputs. Proactive development of this AI-augmented expertise is necessary to shape a future where technology amplifies, rather than attenuates, scientific thought.^14^

## Epistemic challenges and opportunities

### The AI complacency problem

Unlike previous technological transitions, GenAI creates a qualitatively different relationship between scientists and their tools.^15^^,^^16^ When researchers delegate central scientific tasks to an LLM—literature reviews, code generation, methodology design—they engage in something far more consequential than traditional cognitive offloading.^17^ This is problematic, as such tasks have historically been the primary mechanisms through which scientific expertise develops.

Recent studies document concerning patterns of overreliance and deskilling in human-AI collaboration.^18^ Professionals using AI writing assistants progressively disengage from content, initially scrutinizing AI outputs but gradually accepting them with diminishing critical evaluation^11^—a pattern particularly consequential for technical material. The effortful retrieval that consolidates knowledge, the productive struggle that drives expertise development, and the incubation periods that foster creative insights are all potentially compromised.

The problem extends to epistemic authority: how humans calibrate trust when interacting with AI systems. Users begin with high initial trust based on novelty or perceived sophistication but often fail to develop the context-dependent trust calibration that skilled AI use requires.^19^ GenAI produces outputs that appear authoritative and comprehensive yet may contain subtle inaccuracies or “hallucinations” that demand verification and domain expertise.^20^^,^^21^ The behavioral mechanisms behind skill atrophy operate as a self-reinforcing cycle: when AI consistently provides satisfactory output, the perceived cost of verification increases, leading to progressive disengagement.^22^

This cycle reflects ritualized practices in digital literacy—habituated, unreflective workflows that users develop to manage cognitive load.^23^ GenAI amplifies these dynamics through three mechanisms. First, unlike search results that present fragmented information requiring synthesis, AI outputs appear as complete, authoritative arguments that simulate finished expert reasoning. Second, their conversational, human-like presentation masks the statistical nature of their construction, making verification feel unnecessary. Third, the immediate satisfaction of receiving comprehensive-seeming answers accelerates ritual formation.^24^ Indeed, users consistently overestimate an LLM’s accuracy and are swayed by superficial heuristics, such as the length of an explanation, rather than its substance.^25^

Ritualized acceptance of AI authority manifests distinctively across scientific domains. Programming proficiency appears particularly vulnerable, as code-completion tools generate entire functions with minimal human input. Observational studies reveal maladaptive habits: students fall into unproductive cycles of submitting incorrect AI-generated code and asking it to fix its own errors rather than engaging in debugging and reasoning themselves.^26^ Yet, not all tasks are epistemically equal (Table 1). Losing fluency in syntax or boilerplate code differs from losing capacity for algorithmic reasoning and problem decomposition. Strategic offloading of peripheral tasks—rule-based procedures with verifiable outputs such as formatting and routine syntax—frees cognitive resources for core work requiring interpretive judgment, where errors are subtle and demand domain expertise. The epistemic risk lies in delegating novel algorithmic development and debugging strategies that constitute genuine computational expertise.

**Table 1:** Differentiating the epistemic risks of delegating research tasks to AI

Skill category	Example research task	Risk of offloading to AI
Peripheral: procedural	proofreading, formatting citations, boilerplate code	low (rule-based tasks)
Core: integrative	synthesizing literature, interpreting data, theoretical integration	high (risk of superficial synthesis and missed nuance)
Core: generative	novel hypothesis generation, experimental methodology design, identifying design flaws	very high (risk of pseudo-competence and cognitive atrophy)
Meta-skills	evaluating AI outputs, validating claims, iterative prompt refinement	N/A (new AI skills)

Note: peripheral tasks are rule-based procedures with verifiable outputs. Core integrative tasks require interpretive judgment to synthesize information and detect errors. Core generative tasks involve creative problem-solving and conceptual reasoning.

Research design and methodological reasoning—skills honed through years of training—may atrophy similarly if consistently delegated to AI systems.^27^ A key danger of uncritical use thus lies in breeding pseudo-competence: researchers may feel methodologically sophisticated because they can generate plausible-sounding research designs through AI interaction while remaining unable to recognize fundamental flaws or innovative opportunities that require genuine expertise.

This dynamic exemplifies the ironies of automation, where technology intended to reduce workload paradoxically creates new cognitive burdens.^22^^,^^28^^,^^29^ The user’s role shifts from active production to passive evaluation of AI outputs, a mentally taxing task that degrades situational awareness and encourages overreliance. The AI simplifies routine tasks but makes cognitively demanding work—validating methodologies, identifying subtle errors—even harder. Uncritical GenAI use thus fosters superficial competence that falters when faced with genuine scientific complexity.

Perhaps most concerning is the impact on scientific creativity. Innovation emerges from well-developed internal knowledge that reduces working memory demands and enables complex mental schemata.^30^ Uncritical delegation of foundational tasks that build these schemata risks intellectual dependency. True understanding requires encoding information into efficient neural pathways through effortful engagement—the “cognitive friction” of grappling directly with a problem. This friction is not an impediment but rather the catalyst for unexpected connections that drive breakthroughs. If AI increasingly mediates this engagement, we risk narrowing conceptual exploration to paths already optimized in training data—intellectual path dependency that constrains rather than expands scientific horizons.^11^^,^^31^^,^^32^

### Potential benefits

Despite these concerns, GenAI can strengthen certain academic capabilities when thoughtfully integrated into scholarly workflows.

First, learning through exemplars has long been central to academic development. AI systems can function as always-available demonstrations—providing models of writing, code, or analytical approaches that researchers can learn from, though they still require expert evaluation of quality.^3^ This apprenticeship function particularly benefits early-career researchers or those without ready peer feedback. Just as writers develop their craft by studying masterful prose, researchers can learn from AI-generated examples that illustrate effective structures, arguments, or implementations—complemented with tailored explanations.

Second, AI’s accelerated feedback loop addresses a key limitation in traditional skill development. Rather than waiting weeks for feedback, researchers receive immediate, detailed responses that facilitate rapid iteration and compress the learning cycle.^1^ A researcher drafting a methodology section can instantly receive suggestions for improvements—feedback that might otherwise require multiple rounds of peer review. Growing evidence from GenAI-powered intelligent tutoring systems in higher education demonstrates that personalized, self-paced, and interactive instruction can improve learning outcomes and academic achievement.^33^

This function is particularly powerful when conceptualized as a mechanism for enhancing, rather than replacing, human mentorship. GenAI systems excel at providing “extra-recognitive” feedback—non-judgmental, procedural critiques (e.g., of structure, clarity, or code syntax).^34^ By offloading these tasks to an AI, human mentors (such as principal investigators) can focus on “recognitive” feedback: the deep, motivational, and context-rich guidance essential for developing expertise.^35^ Nevertheless, without strategic prompting, AI feedback often emphasizes positive reinforcement over critical evaluation, potentially promoting convergence toward common patterns and homogenizing scholarly voice (Box 1).^36^

Box 1 **Example prompts for AI direction and reflective questions for researcher discernment**

**Part A: Strategic direction**

The following prompts demonstrate how researchers can strategically direct AI systems to promote critical evaluation and intellectual exploration rather than convergence toward common patterns, illustrating the “strategic direction” component of our framework.

Manuscript review and critique: Act as a seasoned expert in [field]. Critically evaluate this manuscript as if reviewing for [journal]. Show potential reasons for rejection and list multiple key reasons. For each key reason, use two or more sub-bullet points to clarify and support your arguments in painstaking detail. Be as specific and detailed as possible.

Divergent research design: Generate 4 fundamentally different experimental approaches to investigate [research question]. For each design, identify its unique strengths, critical limitations, and potential confounds. Explain why a researcher might choose each approach despite its limitations.

Literature synthesis with dissenting views: Summarize the literature on [topic], but specifically emphasize: (1) unresolved contradictions between studies, (2) methodological limitations that undermine confident conclusions, and (3) alternative theoretical interpretations that challenge the dominant narrative.

Methodological vulnerability assessment: You are a hostile reviewer trying to identify fatal flaws in this research design. List every possible threat to validity, confound, or limitation. For each flaw, rate its severity and suggest how it could be empirically tested or addressed.

Alternative interpretation generation: Given these research findings, generate 3–4 competing explanations that could account for the same data. Rank them by plausibility and identify the critical experiments needed to distinguish between them.

**Part B: Critical discernment**

After receiving AI output, researchers should systematically evaluate it using domain expertise and these reflective questions.

• Completeness: what contradictions or dissenting perspectives are absent? Which alternative explanations has the AI failed to surface?
• Methodological validity: what unstated assumptions underlie this AI-generated research design? What threats to validity or confounds require deeper examination than the AI acknowledged?
• Epistemic confidence: does this output’s fluency mask uncertainty or unresolved debates? Where does authoritative presentation obscure genuine scholarly disagreement?
• Verifiability: can every factual claim, statistic, and citation be traced to accessible primary sources? Which assertions demand independent verification before integration into my work?

Third, AI assistance can expand intellectual engagement by reallocating cognitive resources from mechanical to higher-order work. Offloading peripheral tasks—formatting citations, transcribing audio, organizing literature, and generating boilerplate code (Table 1)—frees capacity for activities demanding human judgment: conceptual integration, hypothesis refinement, experimental design, and engaging with broader ideas, methods, and collaborations. For example, AI-assisted reading workflows enable engagement with podcasts, videos, and foreign-language sources without sacrificing depth.^37^ AI-powered voice-to-text tools replace the slow, physically taxing process of typing with high-speed dictation.^38^

Beyond mechanical offloading, LLMs can forecast experimental effect sizes with accuracy matching human experts^39^ and generate high-quality materials indistinguishable from human-created ones.^40^ Yet, these higher-order applications demand strategic direction, rigorous validation, and theoretical integration, not simple delegation. Benefits depend on how researchers approach AI tools. When used with deliberate intention to learn—critically evaluating outputs and treating AI as a collaborative partner rather than a replacement—these systems function as skill amplifiers rather than substitutes.

## Developing AI meta-skills

### Framework

**Figure 1:**
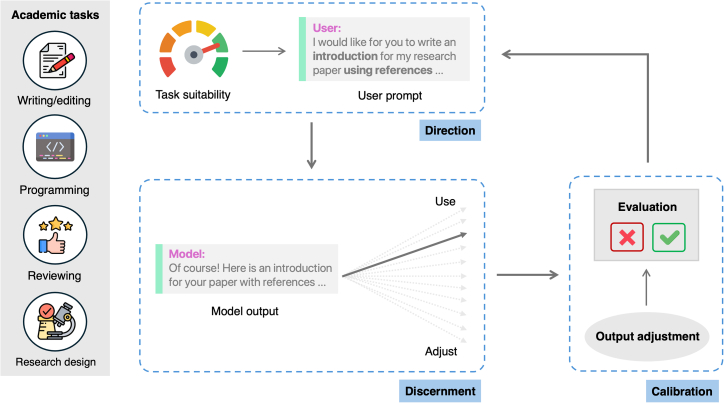
The direction-discernment-calibration framework for AI-augmented expertise The process of maintaining scientific autonomy is a dynamic cycle. (1) Strategic direction: the researcher defines a goal, assesses the task’s suitability for AI delegation (see also Table 1), and crafts prompts to direct the AI. (2) Critical discernment: after the AI generates an output, the researcher evaluates it for accuracy, logical coherence, and bias. (3) Systematic calibration: the researcher makes a judgment: accept, edit, or reject the output. Rejection initiates an iterative refinement loop, returning to the direction stage to refine the prompt. This cycle relies on the researcher’s metacognition, framing expertise as the skillful management of this human-AI collaboration.

Working with AI’s unique capabilities^41^ necessitates specific meta-skills. This involves determining task suitability for AI, discerning outputs, calibrating results, and iteratively resubmitting when needed. It also requires accurately assessing the limits of our knowledge—knowing when to engage with or trust AI versus one’s own knowledge or sources.^42^ This framework emphasizes how scientists strategically guide AI systems while critically evaluating outputs in an ongoing cycle (Figure 1). These interrelated components constitute a novel form of scientific expertise as essential as domain knowledge itself.

Crucially, while building upon critical thinking foundations, AI meta-skills represent a distinct form of intellectual engagement. Traditional critical thinking evaluates static information—assessing claim validity, identifying logical flaws, or weighing evidence. AI meta-skills require dynamic collaboration with generative systems. This collaboration is essential because initial AI outputs often reflect provisional synthesis rather than validated conclusions, which researchers must iteratively guide toward rigorous analysis. Rather than uncritically accepting outputs—or prematurely dismissing the tool—researchers shape AI behavior through iterative prompting and strategic tool selection,^43^ recognize characteristic limitations (such as authoritative-seeming hallucinations), and systematically validate machine-generated content. This procedural, collaborative dimension distinguishes AI meta-skills from conventional critical thinking: researchers become active directors of iterative knowledge generation, requiring metacognition—the ability to monitor and control one’s thinking.^42^^,^^44^

The framework begins with identifying which aspects of scientific work are appropriate for AI delegation versus direct human engagement. This requires understanding AI’s epistemological limitations in specific domains and research contexts.^45^ Researchers must construct prompts with contextual structures and constraints that guide AI reasoning toward scientifically valid approaches. Providing necessary domain knowledge and strategically framing questions elicits comprehensive, well-reasoned responses rather than simplistic answers. This includes divergent prompting, where researchers guide AI systems to explore multiple alternatives, preventing premature convergence on seemingly optimal but potentially limited solutions and fostering intellectual exploration.

Following strategic direction, users must identify subtle inaccuracies, conceptual flaws, or reasoning errors in authoritative-seeming AI outputs.^20^ This “discernment” dimension is particularly crucial where one lacks sufficient expertise. For instance, AI systems often exacerbate citation inequality, disproportionately recommending highly cited papers and potentially skewing the representation of scholars from different backgrounds.^46^ Researchers must develop assessment skills to evaluate AI-suggested research approaches, understanding how generative models represent scientific methodologies and recognizing that AI suggestions reflect statistical patterns rather than causal or theoretical understanding.

Following discernment, users calibrate the generated response based on content suitability and domain expertise. If unsatisfied, users reevaluate the selected model or tool and resubmit with refined prompting.

These components operate in a continuous feedback loop where direction shapes what needs discernment and discernment informs subsequent direction. This dynamic interaction constitutes a new scientific literacy that bridges human cognitive capacities with machine intelligence.

These meta-skills develop through practice with AI systems—much as musical or athletic expertise emerges through deliberate engagement—with observable behavioral indicators that differentiate novice from expert use. Strategic direction manifests through iterative prompt refinement that produces measurable improvements in output relevance and accuracy.^47^ Critical discernment operates through systematic verification protocols—cross-referencing claims against primary sources, interrogating output biases, articulating reasoning flaws—that reliably detect errors overlooked by less experienced users. Systematic calibration emerges through human override decisions that maintain alignment with domain standards, distinguishing plausible-but-flawed AI content from genuinely valid analysis. These behaviors reflect general scientific reasoning applied to AI collaboration.

### Meta-skills in practice

The following cases from recent research demonstrate how these meta-skills operate across diverse domains, revealing both sophisticated applications and critical failures. Each domain illustrates the iterative cycle of strategic direction, critical discernment, and systematic calibration that defines effective human-AI collaboration. Empirical evidence suggests that successful AI integration depends critically on existing domain expertise, with AI systems producing subtle but fundamental errors that non-experts consistently fail to detect.^48^ These cases validate why strategic direction, critical discernment, and rigorous calibration represent essential capabilities rather than optional enhancements to traditional research skills.

### Literature analysis, ideation, and writing

Literature analysis exemplifies how meta-skills operate at the level of tool selection and workflow design. Strategic direction requires distinguishing between general-purpose chatbots—which often lack access to paywalled articles and fabricate citations (particularly without internet access)—and specialized, domain-specific tools designed for scholarly tasks (e.g., NotebookLM).^49^ Sophisticated direction involves choosing appropriate instruments for specific research functions rather than defaulting to familiar AI systems.

Critical discernment becomes essential when evaluating AI-generated literature summaries, as researchers must cross-reference summaries with original sources and distinguish genuine scholarly consensus from algorithmic artifacts.^50^ Effective calibration requires systematic validation that cannot be delegated—breaking complex analyses into manageable components for individual verification rather than accepting AI-generated output wholesale.^51^ These verification processes represent irreducible intellectual labor defining scholarly competence.

Ideation reveals that strategic direction involves assigning specific roles to AI systems—designer, writer, interviewer, or actor—depending on research needs.^52^ However, discernment requires recognizing that current models excel at incremental refinements, while conceptual breakthroughs still require human insight.^31^

Academic writing demonstrates this pattern through systematic collaboration frameworks. Strategic direction operates through a two-stage model: AI-inspired phases for expansive tasks such as brainstorming and structural organization, followed by AI-assisted phases for focused refinement such as drafting and language polishing.^3^ Critical discernment becomes essential to avoid de-skilling over time, requiring verification of AI-generated content and recognizing that essential elements—insight, originality, and creativity—cannot be fully replicated by current systems. Effective calibration employs human-in-the-loop editing where writers maintain ultimate authority, iteratively integrating or discarding AI suggestions while tracking contributions for transparent disclosure.

### Natural sciences

Examination of specific domains reveals how meta-skills adapt to different validation systems. In pure mathematics, researchers demonstrate strategic direction by deploying AI systems to generate novel conjectures from large-scale pattern analysis.^53^ The critical advance lies in calibration: recent systems translate AI-generated insights into formal proof languages such as Lean, enabling definitive logical verification.^54^ When an AI proposes a conjecture, researchers exercise discernment using the “Birch test”—evaluating whether the claim is automatic, interpretable to human mathematicians, and non-trivial. The cycle continues iteratively: failed verification prompts researchers to refine their direction by adjusting axioms, constraints, or problem formulation.

In experimental sciences, calibration operates through fundamentally different mechanisms. Chemistry and materials science employ AI for inverse design: researchers specify desired properties—conductivity, stability, and cost—and direct AI systems to explore vast chemical spaces for candidates.^55^ Strategic direction requires translating scientific objectives into computational constraints. For example, in solid-state battery electrolytes, researchers direct AI to optimize ionic conductivity while maintaining thermal stability. Critical discernment becomes essential when evaluating AI-proposed structures: researchers must assess synthetic feasibility, identify potential side reactions the model ignored, and recognize when structures violate chemical principles despite satisfying computational criteria.

Unlike mathematics, calibration here demands experimental validation—synthesizing compounds and measuring actual properties. This multi-stage verification process, combining computational screening with laboratory testing, exemplifies domain-specific calibration. Biology similarly employs AI agents to design CRISPR experiments and predict gene regulation, but ultimate validation requires wet-lab confirmation rather than formal proof.^55^

### Social sciences

Social scientists employ qualitative interviews, quantitative surveys/experiments, and mixed-methods designs, each requiring different AI engagement strategies.

In qualitative research, strategic direction manifests through careful task decomposition. Researchers direct LLMs to transcribe interviews, perform initial thematic coding, or analyze large text corpora—tasks traditionally requiring weeks of manual labor.^56^ Critical discernment becomes essential when evaluating AI-generated codes: without human-provided examples, LLMs produce generic themes that miss contextual nuance.^57^ One study found moderate-to-strong alignment between human and AI coding only after researchers supplied their own coding schemes, demonstrating the importance of direction quality.^58^ Calibration operates through validation against human-coded holdout samples, ensuring augmentation rather than replacement of interpretive expertise.^59^

Quantitative methods reveal different patterns. In finance and economics, strategic direction alternates between “human-driven exploration” (leveraging LLMs for ideation and question structuring) and “data-driven exploration” (using AI for pattern detection).^60^ Contextual assessment determines appropriate engagement modes: conceptual studies benefit from human-driven ideation, while empirical analyses require data-driven detection.

Critical discernment becomes evident when interpreting AI-detected patterns in trading data and social media sentiment, requiring integration into theoretical frameworks.^61^ Survey research demonstrates additional challenges: while LLMs can draft questionnaires or suggest statistical models, researchers must verify that recommendations align with theoretical constructs and sampling assumptions.^62^ Calibration operates through iterative refinement, ensuring continuous human validation and theoretical integration.

Mixed-methods research exemplifies integrated meta-skill deployment.^63^ When analyzing both survey data and interview transcripts, strategic direction requires separate AI engagement for each modality before synthesis. AI can summarize statistics and generate themes but requires researcher oversight at each step: providing coding examples for qualitative data, selecting appropriate statistical tests for quantitative analysis, and validating integration logic. This staged approach prevents errors in either strand from compromising interpretation.

Psychological science presents the most complex expression of these patterns, where meta-skills confront challenges in literature synthesis, experimental design, statistical analysis, and participant simulation. A comprehensive case study reveals how meta-skills operate throughout an entire AI-assisted project.^64^ Researchers employed strategic direction through a drill-down approach, iteratively prompting AI to move from broad areas (“digital consumption and mental health”) to specific hypotheses about, for example, “ethical fatigue.”

However, constant discernment proved essential: AI-generated literature reviews were unusable due to hallucinated citations and an inability to access paywalled journals, experimental designs contained fatal confounds requiring reconstruction, and statistical analyses appeared plausible while containing major errors. Calibration required substantial human override across all research phases.

Advanced applications require both sophisticated direction and discernment. Researchers now direct LLMs to generate personalized experimental stimuli, tailoring persuasive messages to participants’ traits or creating real-time dialogues to challenge misinformation.^65^ Yet, these applications reveal prompt fragility—minor instruction changes can produce dramatically different outputs, requiring researchers to discern which variations reflect experimental manipulations and which reflect algorithmic artifacts.

Effective calibration employs domain-specific validation: comparing AI coding against holdout self-reports for internal states, holdout human ratings for observational measures, and predictive accuracy for behavioral outcomes.^66^ When researchers use LLMs to simulate human participants, calibration requires recognizing that outputs represent token predictions rather than cognition, demanding systematic validation against actual human data.^67^

### Framework validation

These domain-specific cases validate our theoretical framework while revealing its practical complexity. The meta-skills required for effective human-AI collaboration must be deliberately cultivated rather than assumed to emerge from AI exposure alone. Without such cultivation, researchers risk the progressive skill atrophy described earlier, becoming dependent on technologies they cannot critically evaluate or strategically direct. The consistent pattern across domains—AI augmenting human capabilities within specific constraints rather than replacing human judgment—underscores that these meta-skills are essential for maintaining scientific autonomy as AI becomes ubiquitous.

### Repositioning AI for academic research and education

Rather than positioning AI as either a threat or a savior, we must develop forward-looking approaches that harness its benefits while mitigating risks.^68^ The following principles and stakeholder-specific interventions can guide this balanced integration.

(1) Designing for complementarity: AI developers serving academic markets should prioritize interfaces that promote active engagement over passive consumption.^69^ This may include presenting multiple alternative approaches, explicitly highlighting uncertainties, or requiring substantive user input before generating complex outputs. Systems could integrate literacy-enhancing components—automated confidence indicators, verifiable citation sources, and interactive elements prompting critical evaluation before acceptance.
(2) Research-centric AI literacy development: understanding how and when to integrate AI with one’s own knowledge and recognizing the consequences of overreliance are essential. Yet, most researchers report lacking guidance and training, preventing optimal (and responsible) AI use.^70^ Academic bodies should develop critical AI literacy among users, framing AI engagement as a catalyst for skill development, not merely productivity enhancement. This literacy must extend to working practices: fact-checking outputs and identifying tasks unsuitable for delegation due to ethical and security risks, including intellectual property and data confidentiality (e.g., selecting secure AI systems that explicitly guarantee user data will not be used for model training).^71^

These principles require operationalization through specific stakeholder interventions.

(1) For funding agencies: allocate a percentage of major research grants toward discipline-specific AI literacy, including direction-discernment-calibration training. Fund research examining how AI adoption impacts research processes, skill development (including potential atrophy), and scientific creativity across disciplines and career stages. Require grant proposals to include explicit AI integration strategies and usage disclosure.
(2) For universities: integrate dedicated AI direction-discernment-calibration curricula into existing graduate research methods, statistics, and ethics courses, emphasizing hands-on direction and evaluation of AI-generated content. Implement faculty development programs equipping instructors to teach and model these skills effectively. Create “AI-augmented” research certification programs that assess the ability to critically evaluate and direct AI systems.
(3) For scientific journals: institute standardized, granular disclosure requirements detailing both AI use and human verification procedures. Develop specialized review protocols and train peer reviewers to evaluate AI-assisted manuscripts, identify signs of uncritical AI reliance, and assess reported validation procedures.
(4) For scientific societies: establish discipline-specific guidelines for ethical and effective AI integration. Create working groups to develop domain-relevant open materials for cultivating AI meta-skills and assessing this capacity. Foster community platforms (repositories, forums, and journal space) for sharing best practices, validated prompts, and effective AI workflows.
(5) For individual researchers: cultivate independent practices for self-regulated use. Apply the task vulnerability hierarchy (peripheral versus core) in Table 1 before delegating work. Internalize reflective questions in Box 1, part B, as habits for evaluating AI outputs. Periodically audit AI workflows to distinguish complementary use from substitution. Direction requires protecting intellectual property. Calibration operates as a learning strategy: substantive revisions to AI outputs reflect genuine discernment, while superficial acceptance signals emerging dependency.

These interventions should be supported by systematic research on AI resource inequality. Well-resourced universities can invest in robust AI training, helping faculty and students learn best practices that mitigate skill erosion. In contrast, under-resourced programs may continue maladaptive practices with minimal institutional guidance in critical AI literacy. This emerging AI divide risks stratifying the research landscape and amplifying existing societal disparities.^1^

For instance, recent work shows that the advent of ChatGPT widened the academic productivity gap between male and female researchers, driven by gender differences in adoption and usage patterns.^72^ Such findings underscore that special attention must be given not only to researchers from resource-limited settings but also to how technology adoption intersects with other structural inequities. This includes dedicated funding for training programs and certification opportunities that can be conducted remotely or with minimal technological requirements to prevent further exacerbation of existing inequalities.^73^

Addressing these challenges requires pedagogical approaches that effectively cultivate AI meta-skills across diverse institutional contexts. We propose a situated learning framework that recognizes how direction, discernment, and calibration capabilities develop in practice (Box 2). Drawing on situated learning theory and cognitive apprenticeship models,^74^^,^^75^ this approach develops AI meta-skills through practice within real research contexts rather than mastering abstract principles before engaging with actual tools.

Box 2 **Developing AI direction, discernment, and calibration skills through situated learning**

A fundamental challenge emerges in cultivating AI meta-skills: how can students develop such skills without substantial domain expertise? Direction, discernment, and calibration cannot develop through abstract principles alone but require scaffolded engagement with authentic research materials, where domain knowledge and AI meta-skills develop synergistically.

**Progressive skill development framework**

AI meta-skills develop through four interconnected stages that address the expertise challenge while building transferable capabilities.

Stage 1: foundational pattern recognition. Students develop basic direction skills—framing clear research questions and providing adequate context to AI systems—while identifying quality indicators: fabricated citations with suspicious titles, internal logical contradictions, statistical impossibilities, or claims violating basic constraints. Initial calibration involves binary decisions (accept/reject) rather than nuanced adjustments. These foundational skills transfer across domains because they rely on general reasoning and intuition rather than specialized knowledge.

Stage 2: collaborative meta-skill distribution. Advanced users engage alongside novices in directing AI systems and evaluating outputs, creating distributed cognition systems that leverage complementary capabilities. Experts model sophisticated prompting strategies (direction) and domain-specific evaluation criteria (discernment), while novices contribute fresh perspectives on AI-generated content and alternative calibration approaches. This collaborative approach develops all three meta-skills while circumventing the prerequisite expertise conundrum.

Stage 3: strategic integration development. Students coordinate direction, discernment, and calibration as integrated capabilities rather than separate skills. They develop systematic prompting strategies incorporating verification requirements (strategic direction), apply domain-general validation protocols alongside emerging domain knowledge (contextual discernment), and make graduated adjustments rather than binary decisions (sophisticated calibration). These integrated strategies create reliable frameworks for AI engagement while students simultaneously build domain expertise.

Stage 4: authentic research embedding. Students apply emerging meta-skills within their own research projects, where personal investment and real consequences accelerate skill development. Direction skills develop through actual research questions, discernment through evaluating AI outputs that affect their work, and calibration through iterative refinement in authentic contexts. Faculty guidance ensures quality standards while students develop ownership of their AI collaboration process.

**Implementation across academic career stages**

This framework adapts to different academic development levels. Undergraduates focus on foundational pattern recognition and collaborative meta-skill development through structured exercises. Graduate students emphasize strategic integration and authentic research embedding, applying coordinated meta-skills to thesis work and publications. Faculty model expert-level integration while remaining open to student insights about emerging AI capabilities, recognizing that technological evolution requires continuous meta-skill refinement across all career stages.

**Domain-specific adaptation requirements**

This framework provides foundational principles that must be adapted to specific disciplinary contexts, research cultures, and institutional environments. Effective AI meta-skills manifest differently across fields—no single pedagogical approach addresses the full spectrum of disciplinary requirements and epistemic standards.

Developing these capabilities requires moving beyond abstract principles to concrete engagement with domain-specific challenges. Meta-skills manifest differently in each discipline according to its epistemic standards and validation practices. A chemistry student using AI for experimental design must exercise discernment by identifying chemically implausible reaction pathways or safety hazards that text-trained LLMs cannot recognize, with calibration involving systematic cross-referencing against reaction databases and laboratory safety protocols. A historian using AI to analyze primary sources applies discernment to spot anachronisms, misinterpretations of archaic language, or fabricated quotes, calibrating through verification against archival materials and established historiography. A computational social scientist directing AI to generate sentiment analysis code must discern how the model might misinterpret cultural nuances or perpetuate biases, calibrating against qualitative coding or social theory.

Instructors within each domain must therefore develop tailored guidelines. The framework offers organizing principles rather than prescriptive procedures. Domain experts must translate these stages into specific practices aligning with their students’ research trajectories and professional preparation needs.

These meta-skills and domain knowledge develop through mutual reinforcement rather than prerequisite accumulation. Unlike approaches that dichotomize technical skills such as prompt engineering from human-centered capabilities,^76^ this framework recognizes that robust direction, discernment, and calibration capabilities emerge through direct engagement with the systems researchers will encounter in their careers, making the specificity of AI-focused meta-skill training a pedagogical strength rather than a limitation.^77^

## Concluding remarks

This perspective proposes direction-discernment-calibration as essential meta-skills for AI-augmented research. What does successful AI augmentation require? Our stakeholder roadmap advocates (1) funding agencies supporting discipline-specific meta-skill training and research on AI’s effects on practice; (2) universities integrating direction-discernment-calibration into methods curricula with performance-based certification; (3) journals requiring granular disclosure of AI use and human verification; (4) scientific societies creating validated assessment instruments and best-practice repositories; and (5) individual researchers applying task-vulnerability hierarchies before delegation and auditing workflows to distinguish augmentation from substitution.

Our framework remains conceptual. We lack longitudinal evidence on whether these skills prevent atrophy, performance-based assessments beyond self-reporting, and domain-specific testing across fields with different epistemic standards. Open questions demand attention: can we identify early behavioral markers of maladaptive dependence before atrophy becomes entrenched? How do meta-skill requirements shift as AI systems advance from pattern recognition toward advanced reasoning? What prevents the AI divide from amplifying research inequalities? How do we balance literacy development with technological obsolescence—training for tools that may be superseded within years?

GenAI integration into science is irreversible and accelerating. Whether this produces researchers wielding tools with discernment or serving systems they cannot evaluate depends on deliberate meta-skill cultivation. By pursuing empirical validation while AI practices evolve rapidly, we can shape a future where AI functions as infrastructure for discovery rather than a substitute for thought.

## Acknowledgments

A.S. is supported by an MRC AIM iCASE DTP Studentship (ref. MR/W007002/1). Z.L. is supported by the Yonsei University Research Fund (5355661).

## Author contributions

Z.L. and A.S. wrote the paper.

## Declaration of interests

The authors declare no competing interests.

## Declaration of generative AI and AI-assisted technologies in the writing process

During the preparation of this manuscript, Z.L. used Claude Sonnet 4.5 and Gemini 2.5 Pro to proofread the manuscript, following the prompts described in Lin.^3^ Z.L. reviewed and edited the output and takes full responsibility for the content of this publication.
